# Sikyungbanha-Tang Suppressing Acute Lung Injury in Mice Is Related to the Activation of Nrf2 and TNFAIP3

**DOI:** 10.1155/2020/8125758

**Published:** 2020-03-15

**Authors:** Kyun Ha Kim, Seonju Ahn, Ran Won, Jung Ju Lee, Tae Ho Kim, Jong-In Kim, Jun-Yong Choi, Myungsoo Joo

**Affiliations:** ^1^School of Korean Medicine, Pusan National University, Yangsan 50612, Republic of Korea; ^2^Department of Biomedical Laboratory Science, Division of Health Sciences, Dongseo University, Pusan, 47011, Republic of Korea; ^3^Department of Clinical Korean Medicine, Graduate School, Kyung Hee University, KHU Rd 23, Seoul 02447, Republic of Korea; ^4^Lung Cancer Clinic, Pulmonary Medicine Center, Korean Medicine Hospital of Pusan National University, Yangsan 50612, Republic of Korea

## Abstract

Sikyungbanha-Tang (SKBHT) is a Chinese traditional medicine popularly prescribed to patients with respiratory inflammatory symptoms in Korea. Although the Korea Food and Drug Administration approved SKBHT as a therapeutics for relieving the symptoms, experimental evidence for SKBHT suppressing inflammation is scarce. Here, we presented evidence that SKBHT can suppress inflammation in an acute lung injury (ALI) mouse model and explored the possible underlying mechanisms of SKBHT's anti-inflammatory activity. Single intratracheal (i.t.) injection of SKBHT (1 mg/kg or 10 mg/kg body weight) into mouse lungs decreased prototypic features of lung inflammation found in ALI, such as a high level of proinflammatory cytokines, neutrophil infiltration, and the formation of hyaline membrane, which were induced by a single i.t. LPS (2 mg/kg body weight). When added to a murine macrophage RAW 264.7 cells, SKBHT activated an anti-inflammatory factor Nrf2, increasing the expression of genes regulated by Nrf2. SKBHT suppressed the ubiquitination of Nrf2, suggesting that SKBHT increases the level of and thus activates Nrf2 by blunting the ubiquitin-dependent degradation of Nrf2. SKBHT induced the expression of tumor necrosis factor *α*-induced protein 3 (TNFAIP3), an ubiquitin-modulating protein that suppresses various cellular signals to NF-κB. Concordantly, SKBHT suppressed NF-κB activity and the expression of inflammatory cytokine genes regulated by NF-κB. Given that Nrf2 and TNFAIP3 are involved in regulating inflammation, our results suggest that SKBHT suppresses inflammation in the lung, the effect of which is related to SKBHT activating Nrf2 and TNFAIP3.

## 1. Introduction

Sikyungbanha-Tang (SKBHT) is a Chinese traditional herbal remedy popularly used in Korea as a curative for patients who suffer from inflammatory, respiratory symptoms, including cough, sputum, and fever [1]. Recently, the Korean National Health Insurance System enlisted the prescription of SKBHT as an insurance-covered therapeutics for patients who suffer from inflammatory diseases [2]. Despite the insurance system recognizing the effectiveness of SKBHT, the experimental or clinical evidence for the anti-inflammatory activity of SKBHT is limited; how SKBHT exerts this effect remains less understood. There is a report showing that SKBHT is effective in pneumonia patients and a bronchiectasis patient [1]. In mouse peritoneal macrophages, SKBHT was shown to suppress the production of nitric oxide and proinflammatory cytokines induced by bacterial lipopolysaccharides (LPS) [3]. Given that inflammation is a result of complex interactions among various cell types, it is necessary to assess the effect of SKBHT on inflammation in the context of inflammatory diseases in which numerous cell types are involved.

Acute lung injury (ALI) is a rampant and highly morbid pulmonary disease accompanied by inflammatory reactions and lung tissue damage [4, 5]. Therefore, a high level of neutrophils and tissue damage in the lung incurred by neutrophils is the hallmarks of ALI [4]. The major cause of ALI is a bacterial infection, which is recognized by alveolar macrophages via Toll-like receptor 4 (TLR4) [6, 7]. LPS on bacteria trigger TLR4 signaling that activates NF-κB [8]. Activation of NF-κB eventually leads to the increased expression of cytokines, contributing to the recruitment of inflammatory cells and exacerbating inflammation in the lung [4, 5]. Normally, inflammation is self-limited. One of the mechanisms that restrain inflammatory reaction is via TLR4 signaling [9]. In macrophages active in TLR4 signaling, reactive oxygen species (ROS) are produced and disrupt the function of Keap1, an inhibitor for Nrf2 [10, 11]. The function of Keap1 is to mediate the ubiquitination of Nrf2, making Nrf2 subjected to continual degradation by the 26S proteasome [12]. Therefore, Keap1 inactivated by ROS increases the level of Nrf2, resulting in the activation of Nrf2 [13]. Nrf2 is a transcription factor that increases the expression of genes, including heme oxygenase-1 (HO-1), glutamate-cysteine ligase catalytic subunit (GCLC), or NAD(P)H:quinine oxidoreductase-1 (NQO1) [10, 11]. According to studies with various mouse disease models, Nrf2 plays an important role in relieving ALI, sepsis, and other inflammatory lung diseases [14–16]. Another layer of self-regulation of inflammation can be through tumor necrosis factor *a*-induced protein 3 (TNFAIP3), known as A20, the expression of which is induced by NF-κB [17]. On the one hand, TNFAIP3 inhibits the function of TNF receptor-associated factor 6 (TRAF6), blunting TLR4 signaling [18]. On the other hand, TNFAIP3 affects the activity of receptor-interacting protein 1 (RIP1), inhibiting the signaling from the tumor necrosis factor receptor 1 (TNFR1) [19]. Since these signaling cascades end up activating NF-κB and thus prompting inflammation, the expression of TNFAIP3, in turn, leads to the suppression of NF-κB, inhibiting inflammatory reactions initiated by these signaling cascades.

In this study, we sought obtaining experimental evidence that SKBHT has anti-inflammatory activity. Since the inflammatory reaction is a concerted reaction among various inflammatory cells, we used an ALI mouse model to determine whether SKBHT is capable of suppressing lung inflammation. As possible underlying mechanisms, we examined whether SKBHT activates Nrf2 or TNFAIP3 in macrophages. Based on our findings, we propose that SKBHT could suppress inflammation in ALI mice, the effect of which may be related to Nrf2 and TNFAIP3.

## 2. Materials and Methods

### 2.1. Reagents and Antibodies

SKBHT was purchased from Hankookshinyak Corp., Nonsan, Republic of Korea. The herbal composition in a single dose of SKBHT is denoted in Table 1. MG132 (Sigma-Aldrich Chemical Co., St. Louis, MO, USA), sulforaphane (Sigma-Aldrich), and LPS (*Escherichia coli* O55 : B5) specific to TLR4 (Alexis Biochemical, San Diego, CA, USA) were used. Antibodies against TNFAIP3 (A20), p65 RelA, IκB-*α*, Nrf2, tubulin, *β*-actin, and lamin B were purchased from Santa Cruz Biotechnology (Santa Cruz, CA, USA), except those against HA (H3663, Sigma) and V5 (R960-25, Thermo Scientific, IL, USA).

### 2.2. Assessment of Reactive Oxygen Species

For the measurement of intracellular reactive oxygen species (ROS), flow cytometric analysis was performed as described elsewhere [20]. In brief, RAW 264.7 cells (1 × 10^6^ cells/well) were incubated with 100 *μ*M carboxy-H_2_DCFDA (Thermo Scientific) for 30 min and then analyzed by the BD FACS Canto II (BD Biosciences, San Jose, CA, USA) per the instructions of the manufacturer (Thermo Scientific).

### 2.3. Assessment of Cytotoxicity

Cytotoxicity was determined by using Vybrant® MTT assay kit (Thermo Scientific). Similar to the previous report [20], RAW 264.7 cells (1 × 10^6^ cells) were treated for 16 h with SKBHT dissolved in PBS, and then metabolically active cells were measured by a plate reader (BioTeK, VT, USA) as instructed by the manufacturer's protocol (Thermo Scientific). Live cells were calculated in a percentage against untreated cells. Each assay was conducted in triplicate samples, and measurement was repeated three times.

### 2.4. Acute Lung Injury (ALI) Mouse Model

Animal experiments followed the Guidelines for the Care and Use of Laboratory Animals (the NIH of Korea). The experimental protocol was approved by the IACUC of Pusan National University (protocol number: PNU-2016-1139). Male C57BL/6 mice (7 to 9 weeks old, Jackson Laboratory, Bar Harbor, ME, USA) were used in the study. Mice were anesthetized by Zoletil (Virbac, Carros Cedex, France) prior to intervention. ALI was induced in mouse lungs by administering a single intratracheal (i.t.) injection of 2 mg LPS/kg body weight. Two hours later, two different amounts of SKBHT (1 mg/kg or 10 mg/kg body weight) were similarly injected into the mouse lungs. The amounts of SKBHT were determined, based on the cytotoxicity by SKBHT. At 16 h after i.t. LPS, bronchoalveolar lavage (BAL) was performed with two consecutive injections of 1 ml PBS, and about 300 cells in the BAL fluid were counted in total from three different microscopic fields. The mean number of cells in each field was shown. After being perfused with saline, mouse lungs were inflated with fixatives and embedded in paraffin. Lung sections were prepared in a 5 *μ*m thickness and analyzed by hematoxylin and eosin (HE) staining [21]. At least three different lung slides per mouse were examined in 100x microscopic magnification.

### 2.5. Myeloperoxidase (MPO) Activity

Myeloperoxidase activity in lung homogenate was measured by the myeloperoxidase fluorometric detection kit and the instruction of the manufacturer (Enzo Life Sciences Inc., New York, USA). Lung homogenate was prepared as described elsewhere after perfusion [22]. Data were presented as unit/g tissue.

### 2.6. Maintenance of Cells

RAW 264.7 cells and HEK293 cells were purchased from ATCC (Rockville, MD, USA) and maintained as described elsewhere [20].

### 2.7. Real-Time Quantitative PCR

Total RNA from tissue or cells was isolated with QIAGEN RNeasy®mini kit and the manufacturer's protocol (Qiagen, Germany). One *μ*g of the RNA was reverse-transcribed to cDNA by M-MLV reverse transcriptase (Promega, WI, USA), to which SYBR Green PCR Master Mix (Enzynomics, Daejeon, Republic of Korea) was added. The primers for each gene are as follows: the primers for TNF-*α* were 5′-GGTCTGGGCCATAGAACTGA-3′ and 5′-CAGCCTCTTCTCATTCCTGC-3′; IL-1*β* primers were 5′-AGGTCAAAGGTTTGGAAGCA-3′ and 5′-TGAAGCAGCTATGGCAACTG-3′; IL-6 primers were 5′-TGGTACTC CAGAAGACCAGAGG-3′ and 5′-AACGATGATGCACTTGCAGA-3′; NQO-1 primers were 5′-GCAGTGCTTTCCATCACCC-3′ and 5′-TGGAGTGTGCCCAATGCTAT-3′; HO-1 primers were 5′-TGAAGGAGGCCACCAAGGAGG-3′ and 5′-AGAGGTCACC CAGGTAGCGGG-3′; GCLC primers were 5′-CACTGCCAGAACACAGACCC-3′ and 5′-AGGTCTGCTGAGAAGCCT-3′; and GAPDH primers were 5′-GGAGCCAAAAGG GTCATCAT-3′ and 5′-GTGATGGCATGGACTGTGGT-3′. The thermal reaction was run at 95°C for 10 min, followed by 40 cycles of 95°C for 10 s, 57°C for 15 s, and 72°C for 20 s in a Rotor-Gene Q real-time PCR system (Qiagen). The threshold cycles (Ct) were used to quantify the mRNA expression of the target genes.

### 2.8. Western Blot Analysis

Total and nuclear proteins were prepared by Pierce™ IP lysis buffer and NE-PER™ nuclear extraction kit, respectively, per the manufacturer's protocols (Thermo Scientific). Protein amounts were determined by Bradford (Bio-Rad, Hercules, CA, USA). Equal amounts of proteins were fractionated on NuPAGE gel (Thermo Scientific), which was blotted to PVDF membrane (Bio-Rad). After being blocked with 5% nonfat dry milk for 1 h, the membrane was incubated with primary antibodies at 4°C overnight, and HRP-conjugated secondary antibodies were prepared for 1 h at room temperature. Proteins of interest were revealed by SuperSignal®West Femto (Thermo Scientific). The band intensity of p65 RelA over lamin B was determined by the densitometric analysis software Image J (NIH, Bethesda, MD, USA).

### 2.9. Ubiquitination Assay

Ubiquitination assay was performed as described elsewhere [23]. In brief, HEK293 cells were transfected with plasmids expressing HA-Ub, V5-Nrf2, and FLAG-Keap1 for 48 h. Prior to lysis, cells were treated with 10 *μ*M of MG132 for 3 h. V5-Nrf2 was precipitated by anti-V5 antibody, captured by protein A-sepharose (Thermo Scientific), and analyzed by immunoblotting for HA-Ubiquitin to reveal the ubiquitinated Nrf2.

### 2.10. Statistical Analysis

Paired or unpaired *T*-tests and one-way analysis of variance (ANOVA) tests were used (InStat, GraphPad Software, Inc., San Diego, CA). Data are shown in the mean ± SEM (Std. Error) of at least three measurements. *P* > 0.05 was considered statistically significant.

## 3. Results

### 3.1. ROS Production and Cytotoxicity by SKBHT

**Figure 1:**
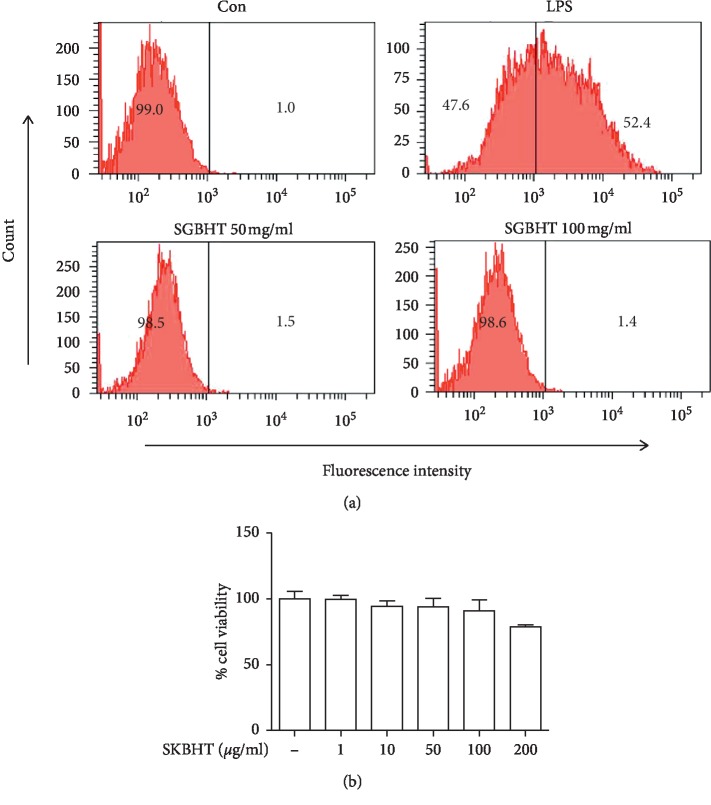
Cytotoxicity by SKBHT. (a) For measuring intracellular ROS induced by SKBHT, RAW 264.7 cells were treated with PBS (Con), LPS (100 ng/ml), or two different amounts of SKBHT (100 *μ*g/ml and 200 *μ*g/ml in PBS) for 16 h Cells were stained with carboxy-H2DCFDA, a ROS indicator, and analyzed by FACS. Cells producing intracellular ROS were shown in percentile at the right side of each panel. A similar experiment was repeated three times, and we obtained similar results. (b) Cytotoxicity due to interference of cellular metabolism by SKBHT was determined by MTT assay. RAW 264.7 cells were treated with increasing amounts of SKBHT for 16 h orcid:0000-0002-5417-1732 Metabolically active cells were determined by measuring the formazan product with a spectrophotometer. Data represent the mean ± SEM of triplicate samples. A similar experiment was repeated twice, and we obtained similar results. No statistical differences between groups were found (ANOVA).

First, we checked whether SKBHT had cytotoxicity. Since excessive production of reactive oxygen species (ROS), which occurs during inflammation, could inflict damage to cells [24, 25], we first tested whether SKBHT induces ROS production. Given that the single dose of SKBHT for an adult (65 kg body weight) is about 3 g, which is equivalent to 46 mg/kg or 46 *μ*g/ml, we treated RAW 264.7 with a high amount of SKBHT (100 *μ*g/ml) for 16 h, and then the production of intracellular ROS was determined by flow cytometer (Figure 1(a)). Results show that, compared with PBS treated (Con), 1 *μ*g/ml of LPS increased the population of RAW 264.7 cells producing ROS (LPS). Unlike LPS, however, SKBHT did not elicit intracellular ROS production (SKBHT 100 *μ*g/ml), which was comparable to the control. To confirm this, we treated RAW 264.7 cells with a higher amount of SKBHT (200 *μ*g/ml). Even in this amount SKBHT did not induce ROS (SKBHT 200 *μ*g/ml), suggesting that SKBHT does induce the production of ROS within RAW 264.7 cells. Next, we tested if SKBHT causes cytotoxicity by interrupting cellular metabolism. MTT assay was performed to address this (Figure 1(b)). Results show that SKBHT up to 100 *μ*g/ml did not exhibit any toxicity to RAW 264.7 cells. Although it appears that 200 *μ*g/ml of SKBHT caused slight toxicity to the cells, this effect was not statistically significant. Together, these results show that less than 200 *μ*g/ml of SKBHT did not cause substantial cytotoxicity in RAW 264.7 cells.

### 3.2. Protective Effect of SKBHT on Acute Lung Injury in Mice

**Figure 2:**
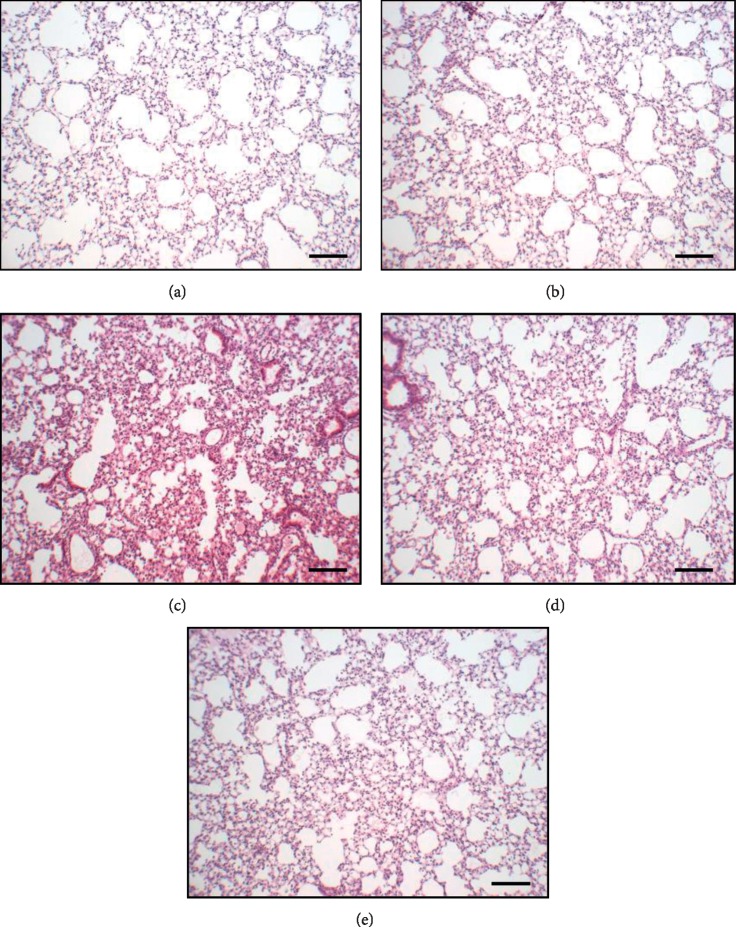
The effect of SKBHT on mouse lung histology. C57BL/6 mice (*n* = 5/group) received a single i.t. injection of PBS (a) or 2 mg/kg b.w. of LPS (c, d, and e). At 2 h after treatments, two different amounts of SKBHT (1 mg/kg b.w. or 10 mg/kg b.w.) were delivered similarly to the lung (d and e, respectively). Lung was perfused and harvested for HE staining. At least three different sections per mouse were analyzed by microscopy. Representative areas of each section are shown (100 magnifications, scale bar = 50 *μ*m).

Since SKBHT is prescribed to patients who suffer from respiratory inflammatory diseases, we determined whether SKBHT suppresses lung inflammation. As one of the most rampant inflammatory pulmonary diseases is acute lung injury (ALI) [4], we tested the anti-inflammatory function of SKBHT using an ALI mouse model (Figure 2). The ALI-like symptom was induced by injecting 2 mg/kg body weight (b.w.) of LPS to the lung of C57BL/6 mice (male, *n* = 5/group) via the trachea. Two hours later, the mouse took a single injection of SKBHT or PBS similarly. At 16 h after the initial intratracheal (i.t.) LPS injection, the anti-inflammatory effect of SKBHT was assessed by HE staining of lung sections. As shown in Figures 2(a) and 2(b), PBS-treated (A) or SKBHT-treated (B) control mice maintained intact airways and normal lung structure. By contrast, LPS-treated mice exhibited characteristics of lung inflammation, including high cellularity and hyaline membrane formation (C), which was, however, suppressed by the single i.t. injection of 1 mg/kg b.w. of SKBHT (D) or 10 mg/kg b.w. of SKBHT (E). These results revealed that SKBHT could reduce inflammation in the lung, suggesting an anti-inflammatory activity of SKBHT.

**Figure 3:**
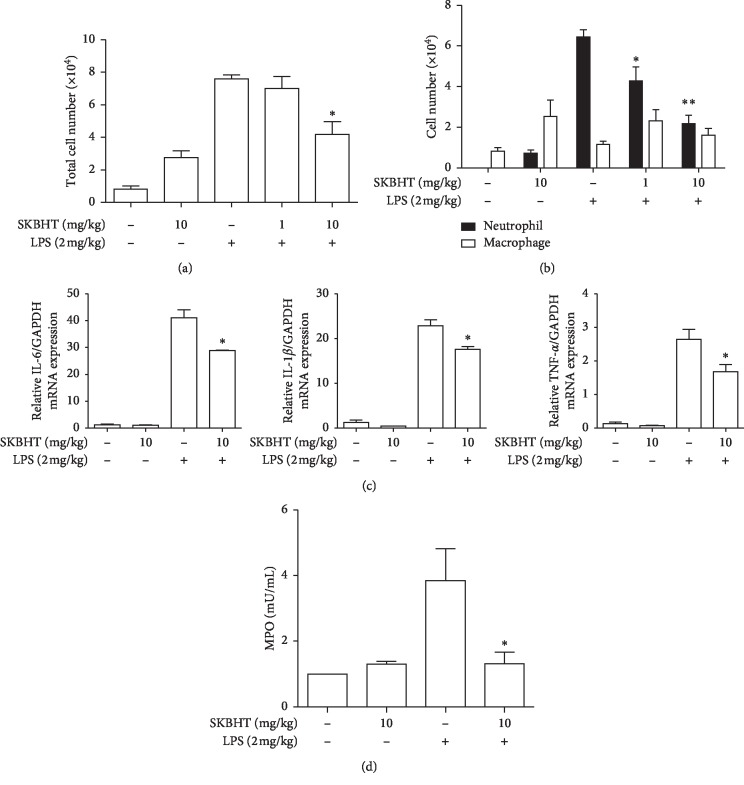
Anti-inflammatory effect of SKBHT on mouse lungs. In bronchoalveolar lavage (BAL) fluid, inflammatory cells were counted after stained. Total cells (a) and macrophages and neutrophils (b) were scored. Lung tissue was homogenized, from which total RNA was extracted for qPCR analysis (c) and MPO activity was measured by ELISA (d). Data are shown in the mean ± SEM of three different samples in each group. ^*∗*^*P* and ^*∗∗*^*P* were less than 0.05, compared to the LPS only (post-ANOVA comparison with Tukey's post hoc test).

To corroborate our findings, we carried out bronchoalveolar lavage (BAL) and analyzed the cells infiltrated to the lung by using the BAL fluid. As shown in Figure 3(a), LPS treatment increased the number of cells in the lung (3^rd^ column), which was significantly diminished by 10 mg/kg b.w. of SKBHT. Differential cell counting (Figure 3(b)) showed that the high cellular infiltration was mainly due to neutrophils (3^rd^ closed column). The infiltration of neutrophils was significantly suppressed by 1 mg/kg b.w. or 10 mg/kg b.w. of SKBHT (4^th^ and 5^th^ closed columns), while the change in macrophages was statistically insignificant (open columns). Since the cellular infiltration to the lung is correlated with the production of proinflammatory cytokines in the lung [9], we determined the levels of such cytokines, IL-6, IL-1*β*, and TNF-*α*, by a quantitative real-time PCR (qPCR) of the mRNA extracted from lung tissue. Consistent with the results in Figure 3(b), SKBHT significantly suppressed the mRNA expression of those cytokine genes in the lung tissue (Figure 3(c)). To further confirm the effect of SKBHT on neutrophils, we measured the myeloperoxidase (MPO) activity that is mainly attributed to neutrophils [1, 26]. As shown in Figure 3(d), SKBHT significantly suppressed MPO activity (4^th^ column), which was induced by LPS treatment (3^rd^ column). Together, these results suggest that SKBHT suppresses lung inflammation in the LPS-induced ALI mice.

### 3.3. SKBHT Activates Nrf2

**Figure 4:**
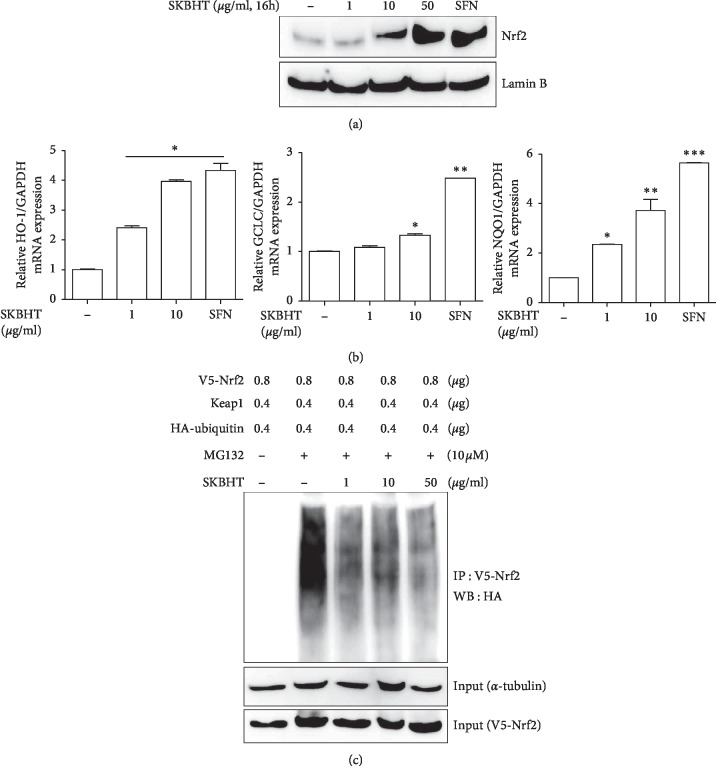
Activation of Nrf2 by SKBHT. (a) RAW 264.7 cells were treated with indicated amounts of SKBHT for 16 h or sulforaphane (5 *μ*M) as a positive control for the activated Nrf2. Nuclear proteins were extracted and analyzed by immunoblotting for the nuclear, active form of Nrf2. The membrane for Nrf2 immunoblotting was stripped and reblotted for lamin B as a loading control. Shown is representative of three independent analyses. (b) Total RNA was analyzed by qPCR for GCLC, HO-1, and NQO-1. Data represent the mean ± SEM of three independent measurements. ^*∗*^*P*, ^*∗∗*^*P*, and ^*∗∗*^*P* were less than 0.001, compared with untreated controls (post-ANOVA comparison with Tukey's post hoc test). (c) HEK 293 cells were transfected with plasmids encoding V5-Nrf2, HA-Ub, and Keap1 for 48 h The transfected cells were treated with or without indicated amounts of SKBHT for 16 h V5-Nrf2 was precipitated by an anti-V5 antibody and analyzed by an anti-HA antibody to reveal the ubiquitinated V5-Nrf2. V5-Nrf2 and tubulin in cell lysate were immunoblotted for inputs.

We attempted to decipher underlying mechanisms for SKBHT to suppress inflammation in the lung. Since Nrf2 is considered a central anti-inflammatory factor [27], we tested whether SKBHT activates Nrf2 to suppress inflammation. First, we examined whether SKBHT activates Nrf2 in cells. RAW 264.7 cells were treated with various amounts of SKBHT for 16 h. Since Nrf2, once activated, moves in the nucleus [11], the nuclear fraction of cells was prepared and analyzed by immunoblotting. As shown in Figure 4(a), similar to the cells treated with sulforaphane (5 *μ*M), an Nrf2 activator [13], SKBHT alone increased the level of the nuclear Nrf2. Since Nrf2 is a transcription factor, we examined whether SKBHT activating Nrf2 is correlated with the mRNA expression of the Nrf2-dependent genes. Total RNA was extracted from the variously treated cells and analyzed by qPCR for the expression of prototypic Nrf2-dependent genes such as HO-1, GCLC, and NQO-1 [10, 11]. Results show SKBHT increasing the expression of those genes (Figure 4(b)). Together, these results suggest that SKBHT activates Nrf2.

To understand how SKBHT activates Nrf2, we examined whether SKBHT dysregulates the ubiquitin-dependent degradation of Nrf2, a major regulatory mechanism of Nrf2 function [28], increasing the level of Nrf2. HEK 293 cells were transfected with plasmids encoding V5-Nrf2, Keap1, and HA-ubiquitin for 48 h and then treated with SKBHT for 16 h. Prior to cell lysis, MG 132, a proteasome inhibitor [29, 30], was added to block the degradation of ubiquitinated proteins. V5-Nrf2 was precipitated by an anti-V5 antibody and analyzed by an anti-HA antibody (HA-tagged ubiquitin) to determine the degree of Nrf2 ubiquitination. As shown in Figure 4(c), Nrf2 was strongly ubiquitinated and shown smeared on the blot (2^nd^ lane), which was undetectable if not treated with MG 132 (1^st^ lane), suggesting the degradation of Nrf2 in the presence of Keap1. When added to the transfected cells, SKBHT decreased the levels of the ubiquitinated Nrf2 (3^rd^ to 5^th^ lanes). These results suggest that SKBHT interrupts Nrf2 ubiquitination, contributing to the increase of Nrf2.

### 3.4. SKBHT Activates TNFAIP3

**Figure 5:**
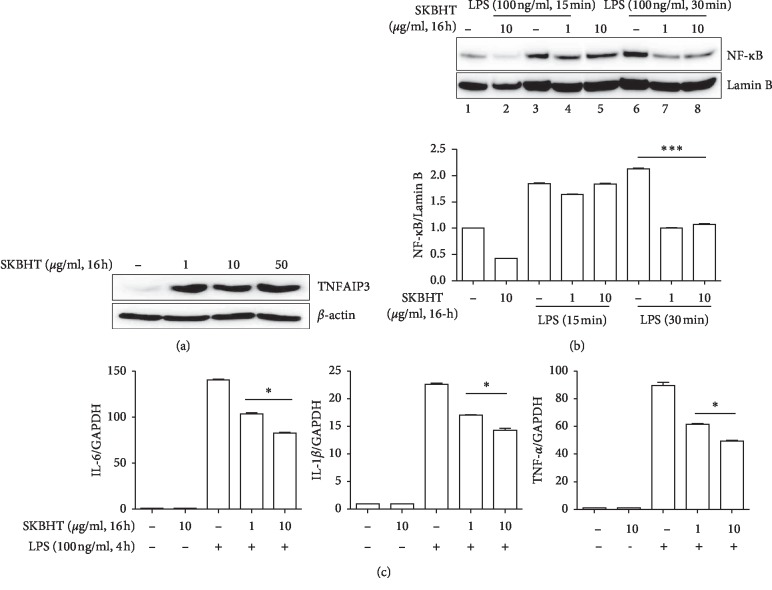
Activation of TNFAIP3 and suppression of NF-κB by SKBHT. (a) RAW 264.7 cells were treated with indicated amounts of SKBHT for 16 h from which the expression of TNFAIP3 was analyzed by immunoblotting. The membrane of TNFAIP3 was stripped and reblotted for *β*-actin. (b) RAW 264.7 cells pre-treated as in (a) were treated with LPS (100 ng/ml) for 15 min or 30 min, from which nuclear proteins were extracted. p65 RelA of NF-κB in the nucleus was analyzed by immunoblotting. The membrane was stripped and reblotted for lamin B for nuclear protein controls. The band intensity of p65 RelA and lamin B was measured three times by Image J The relative levels of p65 (NF-κB) over lamin B are shown in columns. ^*∗∗∗*^*P* was less than 0.001, compared with the LPS only (post-ANOVA comparison with Tukey's post hoc test). (c) RAW 264.7 cells were treated with SKBHT for 16 h and subsequently with LPS for 4 h Total RNA was extracted and analyzed by qPCR for IL-6, IL-1*β*, and TNF-*α*. Data represent the mean ± SEM of three independent measurements. ^*∗*^*P* was less than 0.05, compared with the LPS-treated (post-ANOVA comparison with Tukey's post hoc test).

Given the numerous chemical constituents in SKBHT, it is conceivable that SKBHT exerts its anti-inflammatory function in multiple ways. Since numerous inflammatory stimuli via cognate receptors converge to activate NF-κB, we examined whether SKBHT suppresses the activity of NF-κB by inducing TNFAIP3, an ubiquitin-modulating protein that suppresses proinflammatory signaling originating from receptors for IL-1*β*, TNF-*α*, or LPS [31]. Total proteins were extracted from RAW 264.7 cells treated with varying amounts of SKBHT, and the expression of TNFAIP3 was analyzed by immunoblotting. As revealed in Figure 5(a), the expression of TNFAIP3 was induced by SKBHT treatment. Since TNFAIP3 inhibits TLR4 signaling and consequently suppresses NF-κB activation, we examined whether the expression of TNFAIP3 by SKBHT results in the suppression of NF-κB activity. After being treated with SKBHT for 16 h, RAW 264.7 cells were stimulated with 100 ng/ml of LPS for 15 min or 30 min to activate NF-κB. As described above, nuclear proteins were extracted and analyzed by immunoblotting for the nuclear p65 RelA, the active form of NF-κB [32]. As shown in Figure 5(b), LPS induced the nuclear localization of p65 RelA, suggesting the activation of NF-κB (lanes 2, 3, and 6). SKBHT treatment, however, decreased the levels of nuclear NF-κB (lanes 4, 5, 7, and 8), indicating that SKBHT suppresses NF-κB activity. To confirm SKBHT suppressing NF-κB, we examine the effect of SKBHT on the expression of IL-6, IL-1*β*, and TNF-*α*, well-documented NF-κB-dependent genes. RAW 264.7 cells were treated with SKBHT as above and then with 100 ng/ml of LPS for 4 h to induce the expression of the NF-κB-dependent genes. Analysis of total RNA by qPCR shows that SKBHT significantly diminished the mRNA expression of the NF-κB-dependent genes (Figure 5(c)). Together, these results suggest that SKBHT induces the expression of TNFAIP3, resulting in the suppression of NF-κB activity.

## 4. Discussion

Like traditional Chinese medicine, Korean traditional medicine has treated patients with various “Tang” unique regimens that are composed of several medicinal herbs. The long history of prescribing Tang to patients is considered indicative of the efficacy of Tang, even though experimental and clinical data to support it are limited. For a similar reason, the Korea Food and Drug Administration approved SKBHT as a prescription remedy for patients with inflammatory pulmonary diseases. Here, we set out a study to address this issue. We show that SKBHT suppressed acute lung inflammation in an ALI mouse model, suggesting that SKBHT has anti-inflammatory activity. We evidence that SKBHT activated Nrf2 and induced the expression of TNFIP3 in macrophages. Given the roles of Nrf2 and TNFIP3 in suppressing inflammation, we suggest that SKBHT suppresses lung inflammation, which is associated with SKBHT activating Nrf2 and inducing TNFAIP3.

To obtain evidence that SKBHT has anti-inflammatory activity, we chose ALI as a study model because ALI is one of the most common inflammatory lung diseases with high mortality [4]. Our results show that an injection of LPS via trachea recapitulated key features of ALI patients, including a high degree of neutrophil infiltration to the lung, the increase of proinflammatory cytokines, and the formation of the hyaline membrane in lung tissue [9, 33]. When instilled after LPS administration, SKBHT decreased these features of ALI, which was evidenced by the lung histology close to normal, the decreased proinflammatory cytokines, and the suppressed MPO activity. These results support the notion that SKBHT can suppress lung inflammation, which is concordant with one of the expected effects of SKBHT. In experiments, we delivered SKBHT via trachea to the lung, which is unconventional in Korean medicine; the oral route is common. In general, gavaging Korean medicine is known to take a long time to reach the desired effect and needs to be repeated for several days. Although we did not compare the differential effect between the intratracheal and gavage delivery of SKBHT, it appears that a single intratracheal injection of SKBHT was sufficient to suppress lung inflammation. Besides, we would like to determine whether SKBHT has the anti-inflammatory activity indicated by Korean medicine. Our results clearly show that SKBHT had the potential to suppress inflammation. Since SKBHT is broadly prescribed for other inflammatory lung diseases, testing the efficacy of SKBHT using appropriate respiratory disease models is necessary to consolidate the expected functions of SKBHT.

SKBHT comprises ten different medicinal herbs [1], and some of the herbal constituents of SKBHT exhibit anti-inflammatory activity. For instance, Bupleuri Radix has been used to treat various inflammatory diseases, and its major chemical constituents saikosaponins are known to suppress inflammation [34]. *Pinelliae* Rhizoma has been used to relieve cough, phlegm, and vomiting [35]. *Scutellariae* Radix shows anti-inflammatory effects in both acute and chronic lung inflammation animal models [36]. Armeniacae Amarum Semen is frequently used for treating chronic obstructive pulmonary disease in both stable and acute exacerbation statuses in clinical studies [37]. *Aurantii Fructus* and *Zingiberis Rhizoma* showed anti-inflammatory effects in ALI murine models [38]. Furthermore, *Glycyrrhizae* Radix has antitussive and expectorant activities, and glycyrrhizin, its major compound, ameliorates ALI in a mouse model [39]. Based on these studies, it is conceivable that SKBHT has anti-inflammatory activity, and our study provided evidence for it.

SKBHT likely contains innumerable chemicals. Inflammation involves diverse migratory inflammatory and residential cells in an organ where the inflammatory reaction takes place. Thus, even a single chemical could affect multiple cell types simultaneously, contributing to the regulation of inflammation [40]. Considering the multitudes of chemical constituents and cell types, understanding the detailed mechanism for SKBHT to exert its effects could be challenging. Regardless of this, a possible mechanism by which SKBHT suppresses inflammation has been studied. A report shows that SKBHT blocks the degradation of IκB-*α* in peritoneal macrophages [3]. Although no direct evidence was presented for SKBHT to inhibit the NF-κB activity, SKBHT sustaining IκB-*α*, an inhibitor of NF-κB, likely diminishes NF-κB activity, contributing to the suppression of inflammation, given that NF-κB plays a critical role in inflammation [32].

Consistent with this, our results show that SKBHT suppresses NF-κB by inducing the expression of TNFAIP3. Since TNFAIP3 regulates various inflammatory signaling cascades initiated from the receptors for LPS [41], TNF-*α* [42], or IL-1*β* [43], our results suggest the possibility that SKBHT interrupts a range of proinflammatory signaling cascades simultaneously.

In a self-limiting inflammation, inflammatory reaction grinds to a halt once anti-inflammatory processes start. One of such processes is activating Nrf2, a key anti-inflammatory factor. During inflammation, macrophages produce ROS that can inhibit the function of Keap1, a key inhibitor of Nrf2, which mediates the ubiquitination of Nrf2 and thus drives the degradation of Nrf2 [44]. Therefore, inflammatory reaction, in turn, activates the anti-inflammatory factor Nrf2, contributing to curbing inflammation. While Nrf2 activated by ROS contributes to guarding cells against oxidative challenges [45, 46], Nrf2 plays a key role in ameliorating pulmonary inflammatory diseases, including ALI, emphysema, and asthma [16, 45, 47, 48]. Our results show that SKBHT increased the level of nuclear Nrf2, suggesting that SKBHT activates Nrf2. Immunoprecipitation analysis of Nrf2 shows that SKBHT blocked the ubiquitination of Nrf2, suggesting that the interruption of Nrf2 ubiquitination is one of the mechanisms by which SKBHT activates Nrf2. Since SKBHT did not induce ROS production in our experiments, it is feasible that SKBHT activates Nrf2 without mediating ROS [49]. Concordant with these results, SKBHT increased the mRNA expression of Nrf2-dependent genes, such as GCLC, NQO-1, and HO-1. Collectively, these results strongly suggest that SKBHT activates Nrf2.

**Figure 6:**
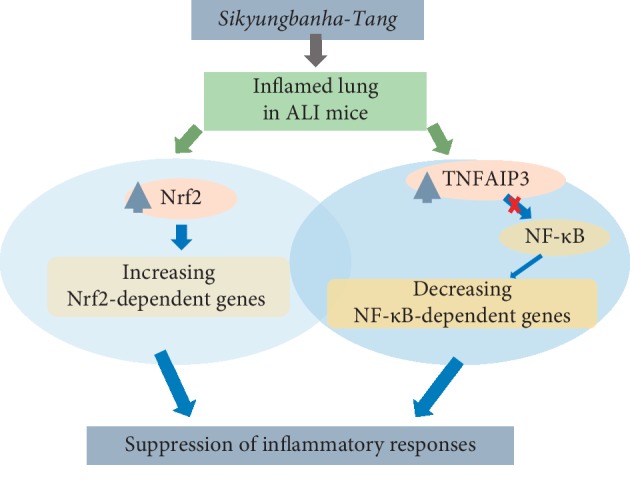
Schematics of proposed mechanisms of SKBHT. When administered to the inflamed mouse lungs, SKBHT could suppress inflammatory reactions in the lungs. Since SKBHT activated Nrf2 and TNFAIP3, which resulted in suppressing NF-κB, in RAW 264.7 cells, we propose that activation of Nrf2 and TNFAIP3 collectively contributes to the suppression of lung inflammation by SKBHT.

Our study has shown that SKBHT suppressed the prototypic features of ALI in a mouse model while activating Nrf2 and TNFAIP3. Given the key roles of Nrf2 and TNFAIP3 in suppressing the inflammatory reaction, it is probable that SKBHT activates Nrf2 and TNFAIP3, which contributes to ameliorating lung inflammation in our ALI mice (Figure 6). It is, however, not clear to what degrees these two factors contribute to the suppressive function of SKBHT in inflammation. The effect of each factor on the function of SKBHT could be addressed by using appropriate knockout mice, and this type of experiment could provide direct evidence that Nrf2 or TNFAIP3 is involved in the efficacy of SKBHT. Also, given the innumerable chemical constituents in SKBHT, not only Nrf2 and TNFAIP3 but also other factors unaddressed in the study could contribute to the anti-inflammatory effect of SKBHT.

In conclusion, notwithstanding these limitations of our study, our results provide evidence that SKBHT has the potential to suppress inflammation acutely occurring in an ALI mouse model, which was consistent with the expected effect of SKBHT. Combined with the findings that SKBHT activated Nrf2 and TNFAIP3, we propose the fact that SKBHT suppressing lung inflammation is associated, at least in part, with Nrf2 and TNFAIP3.

## Figures and Tables

**Table 1:** The herbal composition of SKBHT.

Scientific name	Herbal name	Amount (g)

*Bupleurum falcatum* L.	Bupleuri Radix	0.43
*Trichosanthes kirilowii* MAXIM.	Trichosanthis Semen	0.08
*Pinellia ternate* (THUNB.) BREIT.	Pinelliae Rhizoma	0.31
*Scutellaria baicalensis* GEORGI	Scutellariae Radix	0.56
*Citrus aurantum* L.	Aurantii Fructus	0.39
*Platycodon grandiflorum* (JACQ.)A. DC.	Platycodi Radix	0.54
*Citrus unshiu* MARKOVICH.	Citri Reticulatae Viride Pericarpium	0.31
*Prunus armeniaca* L. var. ansu MAXIM.	Armeniacae Amarum Semen	0.23
*Glycyrrhiza uralensis* FISCH.	Glycyrrhizae Radix	0.14
*Zingiber officinale* ROSC.	Zingiberis Rhizoma Recens	0.03
The total amount per pack (g)		3.02

## Data Availability

The data regarding our findings will be available upon request to the corresponding author.

## References

[1] Kim J.-h., Bhang Y.-h., Do H.-y. (2018). A case report of a patient with bronchiectasis managed with Sikyungbanha-Tang. *The Journal of Internal Korean Medicine*.

[2] Lim B. (2013). Korean medicine coverage in the National Health Insurance in Korea: present situation and critical issues. *Integrative Medicine Research*.

[3] Lee S. E., Shin J. Y., Lee S. H. (2007). Shigyungbanha-Tang exhibits anti-inflammatory effects by inhibiting IκB-*α* degradation in LPS-stimulated peritoneal macrophages. *Journal of Internal Korean Medicine*.

[4] Tsushima K., King L. S., Aggarwal N. R., De Gorordo A., D’Alessio F. R., Kubo K. (2009). Acute lung injury review. *Internal Medicine*.

[5] Martin M. A., Silverman H. J. (1992). Gram-negative sepsis and the adult respiratory distress syndrome. *Clinical Infectious Diseases*.

[6] Beutler B. (2002). TLR4 as the mammalian endotoxin sensor. *Current Topics in Microbiology and Immunology*.

[7] Dreyfuss D., Ricard J.-D. (2005). Acute lung injury and bacterial infection. *Clinics in Chest Medicine*.

[8] Ghosh S., Karin M. (2002). Missing pieces in the NF-κB puzzle. *Cell*.

[9] Bhatia M., Moochhala S. (2004). Role of inflammatory mediators in the pathophysiology of acute respiratory distress syndrome. *The Journal of Pathology*.

[10] Zhang D. D., Lo S.-C., Sun Z., Habib G. M., Lieberman M. W., Hannink M. (2005). Ubiquitination of Keap1, a BTB-kelch substrate adaptor protein for Cul3, targets Keap1 for degradation by a proteasome-independent pathway. *Journal of Biological Chemistry*.

[11] Niture S. K., Khatri R., Jaiswal A. K. (2014). Regulation of Nrf2-an update. *Free Radical Biology and Medicine*.

[12] Voges D., Zwickl P., Baumeister W. (1999). The 26 S proteasome: a molecular machine designed for controlled proteolysis. *Annual Review of Biochemistry*.

[13] Johnson J. A., Johnson D. A., Kraft A. D. (2008). The Nrf2-ARE pathway. *Annals of the New York Academy of Sciences*.

[14] Chan K., Kan Y. W. (1999). Nrf2 is essential for protection against acute pulmonary injury in mice. *Proceedings of the National Academy of Sciences*.

[15] Thimmulappa R. K., Lee H., Rangasamy T. (2006). Nrf2 is a critical regulator of the innate immune response and survival during experimental sepsis. *Journal of Clinical Investigation*.

[16] Sussan T. E., Rangasamy T., Blake D. J. (2009). Targeting Nrf2 with the triterpenoid CDDO-imidazolide attenuates cigarette smoke-induced emphysema and cardiac dysfunction in mice. *Proceedings of the National Academy of Sciences*.

[17] Rothschild D. E., McDaniel D. K., Ringel-Scaia V. M., Allen I. C. (2018). Modulating inflammation through the negative regulation of NF-κB signaling. *Journal of Leukocyte Biology*.

[18] Catrysse L., Vereecke L., Beyaert R., van Loo G. (2014). A20 in inflammation and autoimmunity. *Trends in Immunology*.

[19] Ma A., Malynn B. A. (2012). A20: linking a complex regulator of ubiquitylation to immunity and human disease. *Nature Reviews Immunology*.

[20] Han J. W., Kim K. H., Kwun M. J. (2019). Suppression of lung inflammation by the ethanol extract of Chung-Sang and the possible role of Nrf2. *BMC Complementary and Alternative Medicine*.

[21] Kim K. H., Sadikot R. T., Xiao L. (2013). Nrf2 is essential for the expression of lipocalin-prostaglandin D synthase induced by prostaglandin D2. *Free Radical Biology and Medicine*.

[22] Kim K. H., Kim E. J., Kwun M. J. (2018). Suppression of lung inflammation by the methanol extract of Spilanthes acmella Murray is related to differential regulation of NF-κB and Nrf2. *Journal of Ethnopharmacology*.

[23] Kim K. H., Park H., Park H. J. (2016). Glycosylation enables aesculin to activate Nrf2. *Scientific Reports*.

[24] Shukla V., Mishra S. K., Pant H. C. (2011). Oxidative stress in neurodegeneration. *Advances in Pharmacological Sciences*.

[25] Babior B. M. (2000). Phagocytes and oxidative stress. *The American Journal of Medicine*.

[26] Klebanoff S. J. (2005). Myeloperoxidase: friend and foe. *Journal of Leukocyte Biology*.

[27] Ahmed S. M. U., Luo L., Namani A., Wang X. J., Tang X. (2017). Nrf2 signaling pathway: pivotal roles in inflammation. *Biochimica et Biophysica Acta (BBA)—Molecular Basis of Disease*.

[28] Itoh K., Wakabayashi N., Katoh Y. (1999). Keap1 represses nuclear activation of antioxidant responsive elements by Nrf2 through binding to the amino-terminal Neh2 domain. *Genes & Development*.

[29] Goldbaum O., Vollmer G., Richter-Landsberg C. (2006). Proteasome inhibition by MG-132 induces apoptotic cell death and mitochondrial dysfunction in cultured rat brain oligodendrocytes but not in astrocytes. *Glia*.

[30] Dreger H., Westphal K., Weller A. (2009). Nrf2-dependent upregulation of antioxidative enzymes: a novel pathway for proteasome inhibitor-mediated cardioprotection. *Cardiovascular Research*.

[31] Das T., Chen Z., Hendriks R. W., Kool M. (2018). A20/tumor necrosis factor alpha-induced protein 3 in immune cells controls development of autoinflammation and autoimmunity: lessons from mouse models. *Frontiers in Immunology*.

[32] Zhang Q., Lenardo M. J., Baltimore D. (2017). 30 years of NF-kappaB: a blossoming of relevance to human pathobiology. *Cell*.

[33] Hotchkiss R. S., Karl I. E. (2003). The pathophysiology and treatment of sepsis. *New England Journal of Medicine*.

[34] Li X.-Q., Song Y.-N., Wang S.-J., Rahman K., Zhu J.-Y., Zhang H. (2018). Saikosaponins: a review of pharmacological effects. *Journal of Asian Natural Products Research*.

[35] Nagai T., Kiyohara H., Munakata K. (2002). Pinellic acid from the tuber of Pinellia ternata Breitenbach as an effective oral adjuvant for nasal influenza vaccine. *International Immunopharmacology*.

[36] Shin Y. O., Park C. H., Lee G. H., Yokozawa T., Roh S. S., Rhee M. H. (2015). Heat-processed Scutellariae radix enhances anti-inflammatory effect against lipopolysaccharide-induced acute lung injury in mice via NF-κB signaling. *Evidence-Based Complementary and Alternative Medicine*.

[37] Chen H. Y., Ma C. H., Cao K. J. (2014). A systematic review and meta-analysis of herbal medicine on chronic obstructive pulmonary diseases. *Evidence-Based Complementary and Alternative Medicine*.

[38] Li L., Zhang S., Xin Y. (2018). Role of Quzhou Fructus Aurantii extract in preventing and treating acute lung injury and inflammation. *Scientific Reports*.

[39] Lee S. A., Lee S. H., Kim J. Y., Lee W. S. (2019). Effects of glycyrrhizin on lipopolysaccharide-induced acute lung injury in a mouse model. *Journal of Thoracic Disease*.

[40] Hoebe K., Janssen E., Beutler B. (2004). The interface between innate and adaptive immunity. *Nature Immunology*.

[41] Boone D. L., Turer E. E., Lee E. G. (2004). The ubiquitin-modifying enzyme A20 is required for termination of toll-like receptor responses. *Nature Immunology*.

[42] Tokunaga F., Nishimasu H., Ishitani R. (2012). Specific recognition of linear polyubiquitin by A20 zinc finger 7 is involved in NF-κB regulation. *The EMBO Journal*.

[43] Shembade N., Ma A., Harhaj E. W. (2010). Inhibition of NF-B signaling by A20 through disruption of ubiquitin enzyme complexes. *Science*.

[44] Brüne B., Dehne N., Grossmann N. (2013). Redox control of inflammation in macrophages. *Antioxidants & Redox Signaling*.

[45] Kim K. H., Kim D. H., Jeong N. (2013). Therapeutic effect of Chung-Pae, an experimental herbal formula, on acute lung inflammation is associated with suppression of NF-κB and activation of Nrf2. *Evidence-Based Complementary and Alternative Medicine*.

[46] Sahni S. K., Rydkina E., Sahni A. (2008). The proteasome inhibitor MG132 induces nuclear translocation of erythroid transcription factor Nrf2 and cyclooxygenase-2 expression in human vascular endothelial cells. *Thrombosis Research*.

[47] Rangasamy T., Cho C. Y., Thimmulappa R. K. (2004). Genetic ablation of Nrf2 enhances susceptibility to cigarette smoke-induced emphysema in mice. *Journal of Clinical Investigation*.

[48] Rangasamy T., Guo J., Mitzner W. A. (2005). Disruption of Nrf2 enhances susceptibility to severe airway inflammation and asthma in mice. *The Journal of Experimental Medicine*.

[49] Shah Z. A., Li R.-C., Thimmulappa R. K. (2007). Role of reactive oxygen species in modulation of Nrf2 following ischemic reperfusion injury. *Neuroscience*.

